# HARMONICS: feasibility of a holistic value-based care hybrid programme that maximises clinical outcomes after stroke

**DOI:** 10.1093/esj/aakag016

**Published:** 2026-03-17

**Authors:** Marta Rubiera, Alvaro Garcia-Tornel, Marian Muchada, Francisco Purroy, Joao Sargento-Freitas, Xavier Ustrell, Alejandro Bustamante, Yolanda Silva, Maria Dolors Alsina, Cristina Girao, Gerard Mauri, Sara Bernardo-Castro, Noelia Canela, Elia Dolz, Elisabeth Ortiz, Giorgio Colangelo, David Cano, Marc Ribo, Carlos A Molina

**Affiliations:** Stroke Research Group, Vall d’Hebron Institut de Recerca, Barcelona, Spain; Stroke Unit, Hospital Universitari Vall d'Hebron, Vall d'Hebron Barcelona Hospital Campus, Barcelona, Spain; Stroke Research Group, Vall d’Hebron Institut de Recerca, Barcelona, Spain; Stroke Unit, Hospital Universitari Vall d'Hebron, Vall d'Hebron Barcelona Hospital Campus, Barcelona, Spain; Stroke Research Group, Vall d’Hebron Institut de Recerca, Barcelona, Spain; Stroke Unit, Hospital Universitari Vall d'Hebron, Vall d'Hebron Barcelona Hospital Campus, Barcelona, Spain; Stroke Unit, Hospital Universitari Arnau de Vilanova, Clinical Neuroscience Group, IRBLleida, Universitat de Lleida, Lleida, Spain; Stroke Unit, Department of Neurology, Unidade Local de Saúde de Coimbra, Coimbra, Portugal; Stroke Unit, Hospital Universitari Joan XXIII, Tarragona, Spain; Stroke Unit, Department of Neurology, Hospital Universitari Germans Trias i Pujol, Universitat Autònoma de Barcelona, Badalona, Spain; Stroke Unit, Hospital Universitari Doctor Josep Trueta, Girona, Spain; Gerència Atenció Primària i Comunitària de Girona, Institut Català de la Salut (ICS), Girona, Spain; Stroke Research Group, Vall d’Hebron Institut de Recerca, Barcelona, Spain; Stroke Unit, Hospital Universitari Arnau de Vilanova, Clinical Neuroscience Group, IRBLleida, Universitat de Lleida, Lleida, Spain; Stroke Unit, Department of Neurology, Unidade Local de Saúde de Coimbra, Coimbra, Portugal; Stroke Unit, Hospital Universitari Joan XXIII, Tarragona, Spain; Stroke Unit, Department of Neurology, Hospital Universitari Germans Trias i Pujol, Universitat Autònoma de Barcelona, Badalona, Spain; Stroke Unit, Hospital Universitari Doctor Josep Trueta, Girona, Spain; Stroke Research Group, Vall d’Hebron Institut de Recerca, Barcelona, Spain; NORA Health, Sant Cugat del Vallès, Barcelona, Spain; Stroke Research Group, Vall d’Hebron Institut de Recerca, Barcelona, Spain; Stroke Unit, Hospital Universitari Vall d'Hebron, Vall d'Hebron Barcelona Hospital Campus, Barcelona, Spain; Stroke Research Group, Vall d’Hebron Institut de Recerca, Barcelona, Spain; Stroke Unit, Hospital Universitari Vall d'Hebron, Vall d'Hebron Barcelona Hospital Campus, Barcelona, Spain

**Keywords:** digital health, patient-reported outcomes, post-stroke care, stroke follow-up, value-based healthcare

## Abstract

**Introduction:**

The increasing number of stroke survivors underscores the need for coordinated post-discharge care and systematic outcome monitoring. HARMONICS aimed to provide standardised follow-up, integrating clinician-reported (CROMs) and patient-reported outcomes (PROMs) into a value-based care model.

**Patients and methods:**

Using lean methodology, post-stroke care pathways were mapped, and a harmonised workflow was implemented across 6 comprehensive stroke centres (CSCs) in Spain and Portugal. Consecutive patients discharged home or to socio-rehabilitation facilities with an mRS < 5 were offered participation. Follow-up was conducted via a smartphone app or telephone, enabling bidirectional communication with a case manager for health education, vital sign monitoring and PROMs collection. Feasibility required meeting 4 predefined indicators: inclusion > 60%, 3-month retention > 75%, PROMs completion > 60% and satisfaction > 70% measured by patient-reported experience measurement (PREM). Secondary analyses compared outcomes with historical cohorts.

**Results:**

Between 2022 and 2024, 4209 patients were recruited (40.2% women; median age 73 [IQR 62–81]; 75.6% ischaemic; median admission NIHSS 3 [1–6]; median discharge mRS 2 [1–3]). App use occurred in 59.9% (56% independently). Feasibility was achieved for inclusion (82.8%), retention (84.6%) and satisfaction (72.9%), but PROMs completion was 53.7% at 90 days. Despite mild severity, many reported suboptimal PROMs at 3 months, improving modestly by 1 year. Compared with historical controls, HARMONICS patients showed a better 3-month mRS distribution (OR 1.124; 95% CI, 1.042–1.213; *P* = .0026) and improved PROMs (*P* < .05).

**Discussion and Conclusion:**

HARMONICS is a feasible multicentre value-based follow-up model that promotes education, engagement and self-responsibility, with high rates of healthcare satisfaction reported by stroke survivors.

## Graphical Abstract

**Graphical Abstract:**
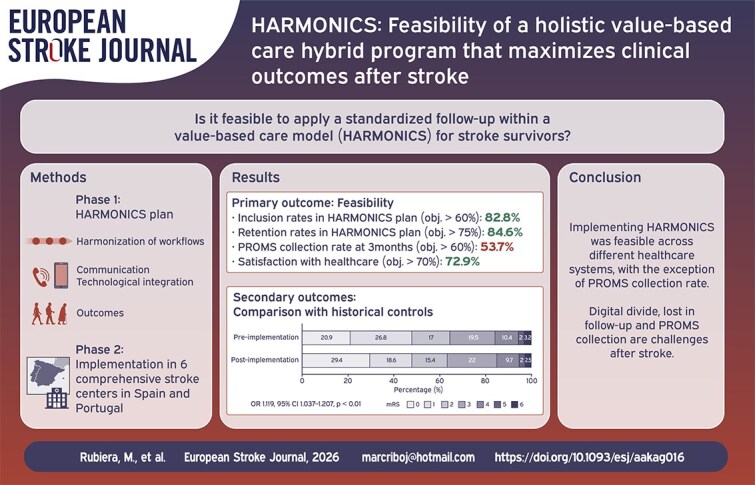

## Introduction

Recent demographic shifts, alongside the increasing incidence of chronic diseases and associated healthcare costs, necessitate a reevaluation of chronic disease management.^1^ Particularly concerning neurological disorders, there has been a notable rise in prevalence and a trend towards long-term chronicity.^2^ Factors contributing to this phenomenon include an ageing population, increased frailty, advancements in diagnostic and therapeutic interventions and enhanced life expectancy. Such trends are particularly evident in developed nations, where demographic ageing is increasingly pronounced.^3^

Stroke serves as a prototypical example of these challenges. Despite considerable advancements in the acute management of stroke, it remains a leading cause of morbidity and mortality globally. In Europe, approximately 1.1 million new stroke cases and 460,000 stroke-related deaths were recorded in the year 2017, accounting for 8% of total mortality. Notably, 9.5 million individuals were identified as stroke survivors.^4^ Upon discharge from medical facilities, nearly 50% of these survivors face disabilities that significantly impair their quality of life, necessitating ongoing post-hospital assistance at the primary care level. This care often encompasses rehabilitation services, social assistance, psychological support and other essential interventions.^5^

Regrettably, despite intensive efforts by both healthcare providers and social support systems to ensure continuity of care, the existing framework remains disjointed. There is a lack of integration across various levels of health care and insufficient coordination between social services and healthcare provisions.^6^ The inadequacy of post-stroke support imposes a significant burden on the families of survivors, who frequently must undertake caregiving roles that disrupt their daily lives and sometimes necessitate the cessation of employment.^7^ Furthermore, the recurrence rate of strokes is alarmingly high, with estimates indicating that 1 in 4 strokes represents a recurrent event. Such recurrences are often a consequence of poor compliance with secondary prevention measures and associated with extended hospitalisation, increased mortality rates and worse levels of disability.^8^

Although adherence to established secondary stroke prevention guidelines has been correlated with diminished recurrence rates,^9^ recent epidemiological studies have failed to indicate a sustained generalised decrease in recurrence events. This observation highlights persistent challenges within healthcare systems.^10^ Consequently, enhanced and comprehensive support during the transition from hospital to home care is essential to avert unplanned readmissions and mitigate related complications.^11^ Additionally, the understanding of patient-reported outcomes (PROMs) remains insufficient, largely attributable to challenges in data acquisition. These factors undermine the principles of value-based healthcare, a patient-centred care model that defines value as patient outcomes relative to the costs of achieving them.^12^ This often results in stroke survivors feeling disconnected from the healthcare system.^13^

In summary, practices that do not adhere to value-based principles contribute to inefficiencies, fragmented care delivery and a lack of continuity after acute stroke episodes. There is a significant deficiency in effective bidirectional communication channels between patients and healthcare professionals, as well as among professionals operating at various levels of care.^14^ In response to these challenges, we aimed to develop HARMONICS, a holistic care plan based on lean methodology,^15^ designed to enhance value-based care and improve post-hospitalisation outcomes for stroke patients. This initiative is envisioned as an initial pilot non-randomised study to assess the feasibility of implementing the proposed plan across diverse comprehensive stroke centres (CSCs) within different healthcare systems.

## Methods

The datasets generated and/or analysed during the current study are not publicly available due to patient confidentiality and data protection regulations but are available from the corresponding author on reasonable request. HARMONICS received ethical approval from the Ethics Committees associated with the 6 participating CSCs, with the coordinator’s committee at Hospital Universitari Vall d’Hebron (HVH) (number: PR(AG)502/2021) granting clearance. All participants, or their respective next of kin when required, provided informed consent prior to inclusion in the study. The HARMONICS study adhered to best practices for reporting non-randomised pilot and feasibility studies, utilising the CONSORT extension^16^ for pilot and feasibility trials (see Table S1). Additionally, due to the observational nature of some study aspects, the STROBE guidelines^17^ were followed (see Table S2).

### Study overview

HARMONICS was financially supported by the European Institute of Innovation and Technology (EIT) Health Europe under grant HARMONICS_EIT-HEALTH-BP2022. This initiative involved collaboration among clinical, administrative, technological and pharmacological partners to establish an integrated approach for delivering high-value stroke care across 5 different CSCs in Catalonia, Spain and 1 CSC in Coimbra, Portugal (https://eithealth.eu/product-service/harmonics/).

The first phase involved a thorough evaluation of current workflows surrounding stroke care, with a particular focus on transitions between hospitalisation and subsequent care phases. The lean methodology, a management approach that maximises patient value by systematically eliminating waste, optimising workflow and promoting continuous improvement, was applied to map and analyse existing clinical pathways.^15^ This mapping involved evaluating the care trajectories of patients with acute stroke both prior to discharge and during post-discharge follow-up across 5 public hospitals in Catalonia, and was complemented by interdisciplinary meetings led by a process engineer. The involvement of neurologists, physiatrists, primary care physicians, social workers, occupational therapists and advanced practice stroke nurses from the different sanitary territories facilitated a comprehensive understanding of care delivery challenges. A parallel assessment was conducted in Coimbra to adapt workflows to the local healthcare context.

The second phase of the study was the progressive implementation of the plan and the evaluation of the results. For this evaluation, predefined clinical outcomes and key performance indicators (KPIs) were established and measured in the second phase (see Table S3). In 1 centre with available data, a comparison with the pre-HARMONICS outcomes was made to define the impact of the implementation.

### Study population

All participating CSCs conformed to national and international guidelines of acute and post-acute stroke care. The HARMONICS plan was implemented alongside standard care protocols as delineated in the European Stroke Organisation recommendations. Following the study initiation at each centre, acute stroke patients scheduled for discharge either to home or socio-rehabilitation facilities were screened for eligibility. The care manager subsequently contacted these patients or their caregivers, provided a study overview and ensured informed consent was obtained prior to inclusion into the HARMONICS programme, facilitating a mobile phone application (Nora, Nora Health, Barcelona, Spain) installation for follow-up when necessary.

Eligible individuals included adults aged 18 years or older who had been hospitalised for acute ischaemic or haemorrhagic stroke or a transient ischaemic attack (TIA), and who were able to offer informed consent directly or via a legal representative. Exclusions encompassed those with social, clinical or functional conditions that might impair 1-year follow-up completion, such as terminal illness, severe disability (defined as an mRS score^18^ of 5), language barriers that hinder communication, instability of residence or contact and plans for follow-up care in another country. Additionally, patients transferred to different acute care hospitals for follow-up were excluded from the study.

### Hypotheses and objectives

We hypothesised that the implementation of a value-based stroke care plan, designated as HARMONICS, would be feasible across various healthcare systems, leading to improvements in clinician-reported outcomes (CROMs) and PROMs, as well as advanced satisfaction in patient experiences with healthcare delivery (patient-reported experience measurements [PREMs]).

The primary objective was to evaluate the feasibility of executing the HARMONICS programme within 6 CSCs operating in 2 distinct healthcare systems. Feasibility metrics, arbitrarily defined by consensus of the multidisciplinary clinical experts participating in the lean evaluation meetings during the first phase of the study, were as follows:

- Inclusion rates of patients in the HARMONICS plan among all admitted stroke patients meeting inclusion criteria (objective: > 60%).
- Retention rates within the HARMONICS plan, quantified by patient engagement through communication with the care manager (≥1 written chat through the app) and/or response rates to surveys for collection of PROMs, maintaining follow-up through the communication app 3 months after the acute stroke event (objective: > 75%).
- PROMs collection at 3 months among all included patients (objective: > 60%).
- Satisfaction with the provided health care, reflected by PREMs (objective: > 70%).

The study’s secondary objectives aimed to assess whether the HARMONICS implementation improved CROMs and PROMs (see Table 1) by comparing them with a historical cohort of prospectively recruited acute stroke patients in the HVH before the initiation of the HARMONICS implementation.^25^

**Table 1:** List of CROMs and PROMs collected in the HARMONICS study.

	**Measure**	**Description**	**Scoring/Interpretation**
	mRS at 90 days^17^	A 7-item ordinal scale measuring global disability.	mRS < 3 indicates functional independence.
	Mortality rate at 90 days and 1 year	Proportion of patients deceased (mRS = 6) at 90 days or 1 year.	mRS = 6 indicates death.
**CROMs**	Stroke recurrence at 1 year	New stroke diagnosis within the first year requiring hospital consultation or admission, occurring at least 24 h after initial discharge. Includes self-reported strokes requiring hospital consultation outside HARMONICs centres.	Documented or self-reported stroke events.
	PROMIS-10^19,20^	Ten-item scale assessing physical function, pain, fatigue, emotional distress, social health and general health perceptions. Produces Global Physical Health (PHY-PROMIS) and Global Mental Health (M-PROMIS) scores.	Scores range 4–20; higher scores = better outcomes. Poor outcome: PHY-PROMIS < 13, M-PROMIS < 11.
**PROMs**	HADS^21^	14-item scale: 7 for anxiety, 7 for depression.	Scores 0–21; higher = worse. ≥10 indicates anxiety or depression.
	EQ-VAS^22^	Self-reported quality of life on a continuous scale.	Range 0–100; higher = better quality of life.
	Fatigue Assessment Scale (FAS)^23^	10-item self-reported scale measuring stroke-related fatigue severity.	Scores 10–50; ≥22 suggests significant fatigue, ≥35 severe fatigue.
	Morisky–Green Levine Medication Adherence Scale^24^	Four-item questionnaire assessing self-reported adherence to pharmacological treatment.	Score ≥ 1 indicates poor adherence.

Abbreviations: EQ-VAS=EuroQol Visual Analogue Scale; HADS=Hospital Anxiety and Depression Scale; PROMIS=Patient Reported Outcomes Measurement Information System; PROMs=patient-reported outcomes.

The primary objective was assessed among all eligible patients from the 6 participating HARMONICS study centres. Secondary objectives were evaluated specifically within the HVH cohort, compared to a historical control group of prospectively recruited acute stroke patients from a previous study of a value-based healthcare plan implemented before HARMONICS was available.^25^ This historical cohort comprised 2160 acute stroke patients admitted to HVH between January 2019 and May 2021. Their inclusion criteria were comparable to those of the HARMONICS study, except that patients with a discharge mRS of 5 were included in the historical cohort but excluded from the present comparative analysis.

Baseline clinical characteristics, stroke subtypes, in-hospital data and CROMs were extracted from structured electronic medical records at each clinical site. For the HARMONICS cohort, PROMs were collected via a communication app or phone and stored on a secure physical server at HVH. For the historical cohort, PROMs were collected via email surveys sent to patients or their next of kin.

### Statistical analysis

Descriptive statistical analyses were performed on continuous variables, employing means and SDs for normally distributed data, and medians with IQRs for non-normally distributed variables. Categorical data were expressed as frequencies and proportions. Univariate comparisons were executed using Fisher’s exact test, Pearson’s chi-squared test, Spearman’s correlation or Mann–Whitney tests, contingent upon variable type and distribution.

The primary outcome, representing the feasibility of implementing the HARMONICS plan, was evaluated against pre-specified thresholds for each metric. A priori sample size calculation was not conducted, as the primary intent was to assess the intervention’s practicality rather than confirm a hypothesis.

For the comparative analysis of secondary outcomes, only patients recruited from HVH in both the HARMONICS-HVH and historical cohorts were included. We performed a complete-case analysis, excluding individuals with missing data on secondary outcome assessments. Stroke recurrence was evaluated as a binary measure across the entire sample.

To address potential confounding, we created 2 distinct propensity-score-matched cohorts: one for mRS at 90 days, and another for PROMs (Patient-Reported Outcomes Measurement Information System [PROMIS]-10, Hospital Anxiety and Depression Scale [HADS], EuroQol Visual Analogue Scale [EQ-VAS] and Fatigue Assessment Scale [FAS]) at 90 days. Propensity scores were determined using logistic regression, incorporating baseline and prognostic factors at discharge such as age, sex, baseline and discharge NIHSS scores, wake-up stroke status, baseline and discharge mRS, stroke subtype (TIA, ischaemic, haemorrhagic), key comorbidities (hypertension, dyslipidaemia, diabetes, smoking, atrial fibrillation, ischemic heart disease), antithrombotic therapies and reperfusion treatments.

Patients were matched 1:1 without replacement using nearest-neighbour “greedy” matching with a calliper of 0.2 SDs of the logit of the propensity score. Balance was assessed using standardised differences, with values ≤ 0.1 indicating acceptable balance. Between-group comparisons used Wilcoxon–Mann–Whitney generalised odds ratios (gOR) for ordinal outcomes, crude odds ratios (ORs) from binomial logistic regression for binary outcomes, and mean differences for continuous measures.

All analyses comparing secondary outcomes between the HARMONICS-HVH and historical cohorts were exploratory and not adjusted for multiple hypothesis testing. Differences are reported with effect measures, 95% CIs and *P*-values without adjustment for multiple hypotheses. All statistical analyses were executed using SPSS version 25 (IBM Corp.) and R (R Foundation for Statistical Computing).

## Results

### Results from the first phase: pre-HARMONICS evaluation and HARMONICS plan design

Lean methodology analysis revealed that, despite procedural variations between Catalonia and Coimbra CSCs, core stroke care principles were similar. After admission to a Stroke Unit, patients receive acute treatment, monitoring and etiological workup, along with assessments of functional, social and clinical needs. Discharge plans address risk factor management, comorbidities and secondary prevention, with follow-up coordinated primarily by primary care teams. It is necessary for either the patient or a caregiver to schedule the primary care visit for this transition.

If rehabilitation or social support needs are identified in-hospital, referrals are made, but significant delays before the first appointment are common. When such needs arise after discharge, patients or caregivers must contact primary care, leading to further delays.

Patients unable to return home may be transferred to rehabilitation or nursing facilities, though care continuity is often fragmented. Hospital–primary care interaction is largely patient-driven.

Neurologist follow-up usually occurs 3 months post-stroke, limiting interim specialist contact. These visits involve non-standardised evaluations of clinical, functional and post-stroke complications. Before the HARMONICS initiative, only 1 of 6 CSCs systematically collected PROMs, using email questionnaires for patients and caregivers.

#### HARMONICS plan

Following the evaluation, the HARMONICS improvement plan was designed to enhance continuity of care through harmonised workflows, improved communication, standardised outcome collection and increased patient education and empowerment, alongside systematic assessment of patient satisfaction.

A hospital case manager (CM) role was introduced, staffed by healthcare professionals with backgrounds in occupational therapy and nursing, with stroke management expertise. Before discharge, the CM contacted the patient and/or next of kin, provided an explanation of the HARMONICS plan, trained them in the use of the communication app and obtained informed consent. To improve continuum of care and increase patient’s trust, the same CM would perform the follow-up of the patients within the HARMONICS plan. A full-time hired CM oversaw approximately 200 patients simultaneously.

The plan included:

- *Harmonisation of workflows*: A predefined schedule for patient–professional contact ensured continuity of care post-discharge. Monitoring non-adherence and complications was carried out via scheduled surveys and direct communication. Specific action protocols allowed expedited responses outside standard visits—for example, sustained high systolic blood pressure prompted telehealth review with the CM and possible treatment adjustments by the neurologist.
- *Communication*: The mobile app and web platform NORA (www.nora.bio) enabled continuous contact between patients or caregivers and CMs through chat, calls and video, alongside personalised education materials.^26^ Patients entered vital signs, received medication reminders and confirmed doses. Alerts were generated on the web platform for abnormal readings, low PROM scores or suspected non-adherence, prompting immediate CM follow-up (see Figure S1).
- *Definition and collection of outcomes:* A set of CROMs, PROMs and PREMs was established based on the International Consortium of Outcomes (ICHOM) Stroke Set recommendations,^27^ with added surveys for adherence^28^ and post-stroke fatigue.^23^ Key performance indicators (KPIs) for the HARMONICS implementation were predefined. A detailed account of KPIs, PROMs and CROMs can be found in Table S3. All patients had a 3-month post-stroke visit for CROMs collection, while PROMs were gathered via the app or phone.
- *Technological integration:* The system generates a secure database hosted at 1 CSC, integrating app data, CM web inputs and electronic health records from all 6 CSCs. This supports continuous KPI monitoring, benchmarking and corrective actions to optimise data collection and overall outcomes (Figure S1).

### Results from the second phase: HARMONICS implementation

#### Study population

**Figure 1:**
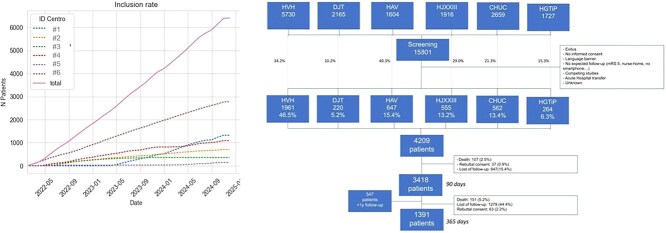
Study flowchart and recruitment progression. Abbreviations: CHUC = Centro Hospitalar e Universitário de Coimbra; DJT = Hospital Universitari Doctor Josep Trueta; HAV = Hospital Universitari Arnau de Vilanova; HGTiP = Hospital Universitari Germans Trias i Pujol; HJXXIII = Hospital Universitari Joan XXIII; HVH = Hospital Universitari Vall d’Hebron.

Beginning in February 2022, a progressive implementation of the HARMONICS initiative and recruitment of patients was conducted across 5 CSCs in Catalonia; in January 2023, the HARMONICS programme was further extended to Coimbra (see Figure 1). The recruitment period for participants was established until November 2024, aligning with the predefined duration of the research grant.

Overall, a total of 4209 patients were enrolled in the HARMONICS study. The baseline clinical characteristics of the study population are detailed in Table 2.

**Table 2:** Baseline clinical characteristics and follow-up summary of the HARMONICS patients.

**Baseline clinical characteristics**	***n* = 4209**
**Age (median, IQR; mean ± SD)**	73 (62–81); 73 **±** 14.0
**Sex, female (*n*, %)**	1693 (40.2)
**Stroke subtype (*n*, %)**	Ischaemic stroke: 3133 (74.5) TIA: 707 (16.8) ICH: 359 (8.5) Other: 10 (0.2)
**Initial NIHSS (median, IQR; mean ± SD)**	3.0 (1.0–6.0); 4.8 **±** 6.0
**Discharge NIHSS (median, IQR; mean ± SD)**	1.0 (0.0–2.0); 1.7 ± 2.7
**Baseline mRS (median, IQR; mean ± SD)**	0.0 (0.0–1.0); 0.8 ± 1.1
**Discharge mRS (median, IQR; mean ± SD)**	2.0 (1.0–3.0); 1.7 ± 1.3
**Discharge destination (*n*, %)**	Home: 3478 (82.6) Socio-rehabilitation centre: 573 (13.6) Nursing-home: 56 (1.3) Acute hospital: 102 (2.4)
**Follow-up summary**	
** Type of initial follow-up (*n*, %)**	NORA app: 2521 (59.9) (56.0 patient) Phone call: 1688 (40.1)
** Type of follow-up at 90 days (*n*, %)**	NORA app: 1586 (37.7) Phone call: 1832 (43.5) Death: 107 (2.5) Rebuttal consent: 37 (0.9) Lost to follow-up: 647 (15.4)
** Type of follow-up at 365 days (excluding 547 patients with less than 1 year of follow-up at the end of the study) (*n*, %)**	NORA app: 815 (28.2) Phone call: 576 (20.0) Death: 151 (5.2) Lost to follow-up: 1279 (44.4) Rebuttal consent: 63 (2.2)
** Number of chats**	Median: 11 (IQR: 4–25) 2052 (81.3%) patients had ≥1 chat
** Number of videos visualised through NORA (median of 9 videos sent per patient)**	Median: 2 (0–8) 1674 (66.4%) patients have seen at least 1 video 736 (29.2%) have seen all sent videos
** Number of documents visualised through NORA (median of 3 documents sent per patient)**	Median: 1 (0–3) 1313 (52.1%) patients have seen at least 1 document 710 (28.2%) have seen all sent documents
** Number of surveys from PROMs collection answered through NORA (median of 27 surveys sent per patient)**	Median: 6 (0–20) 1636 (64.9%) answered at least 1 survey 194 (7.7%) answered all sent surveys
** Number of alerts for medication forgetfulness through NORA**	Median first week: 6 (3–22); 5.1% without any alert Median fourth week: 0 (0–1); 74.9% without any alert
** Rate of surveys for PROMs collection answered (all patients) %**	Median rate of 90 days surveys completion: 53.7 Median rate of 365 days surveys completion: 34.2
** Laboratory tests (3 months) and vitals along follow-up**	Median LDL-cholesterol (at 3 months): 73.5 (56–92) (*n* = 640) Median systolic blood pressure: 127 (118–135) (*n* = 1307) Median diastolic blood pressure: 71 (67–80) (*n* = 1298)
** Recommend attention to family or friend (0–10) (median, IQR)**	10 (8–10); 72.9% scores 9–10
** Degree of satisfaction with the attention at discharge (0–10) (median, IQR)**	10 (9–10); 75.1% scores 9–10
** Degree of satisfaction with the attention after 90 days (0–10) (median, IQR)**	9 (8–10); 65.9% scores 9–10

Abbreviation: PROMs=patient-reported outcomes.

Most patients included in the study did not have any functional disability before the stroke occurrence (as reflected by the median pre-stroke baseline mRS of 0). The majority were recruited following an acute ischaemic stroke, predominantly exhibiting mild severity (median initial NIHSS 3). More than 80% of cases were discharged home, with a median mRS of 2, that improved to 1 after 3 months and 1 year. Table 3 summarises the main clinical and outcome measures (CROMs and PROMs) observed at both 90-day and 1-year follow-ups.

**Table 3:** Main clinician and patients’ reported outcomes after 90 and 365 days, and outcomes evolve along follow-up.

**Outcome**	**90 days (*n* = 4209, % answers)**	**365 days (*n* = 2884, % answers)**	**Wilcoxon test (90–365 days)**	**Evolution (90–365 days)**
**Mortality**	107 (3.5%) *n* = 3063 (73%)	151 (9.3%) *n* = 1622 (56.2%)	---	---
**Stroke recurrence**	---	119 (7.3%) *n* = 1622 (56.2%)	---	---
**mRS (median, IQR; mean ± SD; *n* (%) > 2)**	1.0 (0.0–3.0); 1.6 ± 1.5; 877 (28.7%); *n* = 3063 (73%)	1.0 (0.0–3.0); 1.8 ± 1.8; 530 (32.6%); *n* = 1622 (56.2%)	*P* = .957	0 (0–0) *n* = 1556 67 (4.3%) improve 107 (6.9%) impair
**HADS depression (median, IQR; mean ± SD; *n* (%) > 9)**	2.0 (1.0–6.0); 3.9 ± 4.1; 259 (11.3%); *n* = 2288 (54.5%)	2.0 (0.0–5.0); 3.2 ± 3.8; 88 (8%); *n* = 1076 (37.3%)	*P* < .001 *r* = 0.21	0 (−2–+1) *n* = 994 82 (8.2%) improve 44 (4.4%) impair
**HADS anxiety (median, IQR; mean ± SD; *n* (%) > 9)**	3.0 (0.0–6.0); 4.0 ± 3.9; 234 (10.2%); *n* = 2288 (54.5%)	2.0 (0.0–5.9); 3.1 ± 3.6; 68 (6.3%); *n* = 1076 (37.3%)	*P* < .001 *r* = 0.23	0 (−2–+1) *n* = 927 65 (7.0%) improve 28 (3.0%) impair
**PROMIS-PHY (median, IQR; mean ± SD; *n* (%) < 13)**	15.0 (13.0–18.0); 14.9 ± 3.2; 752 (32.7%); *n* = 2298 (54.6%)	16.0 (14.0–18.0); 15.5 ± 3.1; 200 (18.1%); *n* = 1107 (38.4%)	*P* < .001 *r* = 0.18	0 (−1–+2) *n* = 927 161 (16.5%) improve 95 (9.8%) impair
**PROMIS-M (median, IQR; mean ± SD; *n* (%) < 11)**	13.0 (11.0–15.0); 13.3 ± 3.3; 673 (29.3%); *n* = 2298 (54.6%)	14.0 (12.0–16.0); 13.5 ± 3.3; 183 (16.5%); *n* = 1107 (38.4%)	*P* < .001 *r* = 0.11	0 (−1–+2) *n* = 973 142 (14.6%) improve 85 (8.7%) impair
**FAS (median, IQR; mean ± SD; *n* (%) > 34)**	15.0 (11.0–21.0); 17.3 ± 7.8; 124 (5.4%); *n* = 2279 (54.1%)	16.5 (12.0–24.75); 20.5 ± 10.4; 7 (1.6%); *n* = 420 (14.6%)	*P* = .052	−1.5 (−5–+1) *n* = 360 10 (2.8%) improve 5 (1.3%) impair
**MG (median, IQR; mean ± SD; *n* (%) > 0)**	0.0 (0.0–0.0); 0.1 ± 0.4; 244 (10.2%); *n* = 2403 (57.1%)	0.0 (0.0–0.0); 0.9 ± 0.4; 68 (6.0%); *n* = 1137 (39.4%)	*P* = .196	0 (0–0) *n* = 1006 73 (7.3%) improve 47 (4.7%) impair
**Quality of life (0–100) (median, IQR; mean ± SD)**	71.5 (52.25–85.0); 68.0 ± 24.4; *n* = 1968 (46.8%)	80.0 (60.0–90.0); 73.8 ± 18.6; *n* = 901 (31.2%)	*P* = .002 *r* = 0.11	0 (−10–+10)

Abbreviations: FAS=Fatigue Assessment Scale; HADS=Hospital Anxiety and Depression Scale; MG=Morisky–Green Scale; PROMIS-M=Patient Reported Outcomes Measurement Information System-mental health domain; PROMIS-PHY=Patient Reported Outcomes Measurement Information System-physical health domain.

The mortality rate at 1-year post-stroke was 9.3%, while the recurrence rate was 7.3%. Notably, the median discharge NIHSS score was 1 (IQR 0.0–2.0), and the median mRS score at 3 months was 1 (0.0–3.0). Despite these favourable outcomes, approximately 30% of patients reported poor self-perceived physical and mental health, as measured by the PROMIS-10 scale (see Table 1). Additionally, scores for anxiety and depression on HADS were below the normative threshold in 11% and 10% of cases, respectively (10.4% and 9.1% when excluding individuals with a prior diagnosis of an anxiety-depressive syndrome). Extreme fatigue was reported in FAS by 5% of patients. Furthermore, a positive indication of suboptimal medication adherence, as suggested by the Morisky–Green Scale, was noted in 10% of the participants. The 1-year follow-up of PROMs indicated a statistically significant, albeit modest, improvement in some measures in comparison with those collected at 90 days post-intervention. Most participants reported consistent PROMs over the 90-day and 365-day periods, with the maximum follow-up duration for each patient being 12 months, and a median recruitment duration of 12 months (IQR 10–12).

#### Primary objective: feasibility

The primary objective of this study was to evaluate the feasibility of including patients within the HARMONICS plan, defined by 4 predefined metrics discussed further.

### Inclusion rates in the HARMONICS plan (objective: > 60%)

Throughout the varied recruitment periods, a total of 15,801 patients were admitted to the 6 participating CSCs. Among these, 5084 individuals (32.6%) met the inclusion and exclusion criteria. Notably, 262 patients declined to provide informed consent, and 613 individuals were excluded without stated reasons. Consequently, 4209 participants were integrated into the HARMONICS plan, reflecting a penetration rate of 82.8%.

### Retention rates within the HARMONICS plan (objective: > 75%)

From all the cohort, 2521 (59.9%) patients or next of kin were included in the study as users of the communication app. From them, 2052 (81.3%) engaged with the CMs through at least 1 written communication, while 1636 patients (64.9%) completed at least 1 survey for PROMs collection at the designated 90-day follow-up through the app.

After 3 months, 1586 patients (62.9%) continued their follow-up through the NORA app, and 554 (22.0%) changed to phone communication with the CM by phone. The rate of follow-up visits lost at 3 months was 15.4% (647 patients, 10.9% in patients followed by phone and 15.8% in those followed with the app, *P* < .01), leading to an overall retention rate of 84.6% within the HARMONICS plan.

### Median PROMs collection rate at 3 months (objective: > 60%)

This objective was not fulfilled, as the median PROMs collection of the whole series was 53.7% after 3 months from the index stroke.

### Satisfaction with health care (objective: > 70%)

Notably, the evaluation of patient satisfaction with healthcare services rendered, as indicated by PREM, that 72.9% of patients expressed a willingness to recommend the HARMONICS plan to a relative or friend experiencing a stroke. Satisfaction scores recorded median values of 10 (IQR 9–10) immediately following discharge and 9^8-10^ at 3 months.

Further implementation details regarding the HARMONICS plan are documented in Table 1. Among the 2521 patients monitored through the communication app, 56% independently downloaded and managed the application on their own devices. Notably, individuals using the app independently were more likely to be male (67% vs 50.8%, *P* = .001), substantially younger (median age 66 years [55–75] vs 78 years [72–84], *P* = .002) and exhibited milder strokes (median baseline NIHSS 2 [0–5] vs 4 [1–11]; median discharge NIHSS 1 [0–2] vs 1 [0–4], both *P* < .001).

While retention within the HARMONICS plan demonstrated commendable engagement at 90 days, a notable decline in follow-up rates was observed at 365 days, with nearly 45% of patients lost to follow-up (see Figure 1). Patients who were lost to follow-up presented with lower mRS scores at discharge (median mRS 2 [0–4] vs 1 [0–3], *P* < .001), and the loss to follow-up was significantly higher when patients, as opposed to family caregivers, managed the app/phone answers by themselves (31.2% vs 46.5%, *P* < .001). No significant associations with sex, age or baseline stroke severity emerged in relation to follow-up losses.

Engagement with the communication app was substantial, with each patient averaging 11 written communications with the CM. During the initial week of follow-up, 95% of participants received at least 1 alert regarding medication non-adherence due to forgetfulness; however, by the fourth week, this rate dropped below 25%. More than half of the patients (66%–52%) engaged in health education through the review of individualised videos and informational documents. Approximately 65% of patients completed surveys distributed via the application for PROMs collection at the 90-day follow-up. Communication regarding blood pressure measurement was achieved in 50% of cases through the app, while information on low-density lipoprotein cholesterol levels was documented in only 15% of cases.

#### Secondary objectives (HVH)

Baseline clinical characteristics before and after propensity score matching of the historical cohort and the HARMONICS-HVH cohort are shown in Tables S4 and S5. No significant differences were found between both cohorts after matching. Stroke recurrence was also similar in both cohorts (13.4% in the historic cohort and 13.9% in HARMONICS-HVH [OR 1.05; 95% CI, 0.88–1.24]).

**Figure 2:**
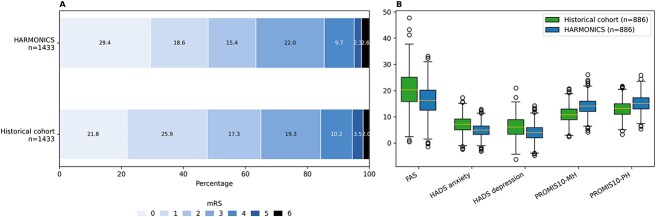
mRS and main PROMs after 3 months from stroke admission in both the historic and HARMONICS-HVH groups after propensity score matching. Abbreviations: FAS = Fatigue Assessment Scale; HADS = Hospital Anxiety and Depression Scale; HVH = Hospital Universitari Vall d’Hebron; PROMIS-10 = Patient Reported Outcomes Measurement Information System (10-item scale); PROMIS-10-MH = PROMIS10 mental health domain; PROMIS-10-PH = PROMIS-10 physical health domain; PROMs = patient-reported outcomes.

The patient subgroup with available mRS scores at 90 days comprised 2167 individuals from the historical cohort and 1480 from the HARMONICS-HVH cohort. Following matching procedures, 1439 individuals from each cohort were compared, revealing no significant differences between the groups (see Table S4). In the matched cohort, a more favourable mRS distribution was detected in the HARMONICS-HVH population: median mRS of 3 (range 2–4) for the historical cohort and 3 (range 1–4) for the HARMONICS-HVH cohort (generalised OR 1.119; 95% CI, 1.037–1.207; *P* < .01). The mortality rates in the historical and HARMONICS-HVH cohorts were 2.4% (34 patients) and 2.5% (36 patients), respectively (OR 1.02; 95% CI, 0.68–1.55, *P* = .6) (Figure 2).

The patient subgroup with available PROMs at 90 days consisted of 1076 in the historical cohort and 963 in the HARMONICS-HVH cohort. Post-matching, 886 individuals from each cohort exhibited no differences in baseline covariates (see Table S5). Subsequent analyses indicated that the HARMONICS-HVH cohort reported superior outcomes compared to the historical cohort, with mean differences of 2.5 points (95% CI, 0.45–4.56; *P* = .01) in the EQ-VAS, 1.25 points (95% CI, 0.84–1.65; *P* < .01) in the HADS-anxiety scale, 1.66 points (95% CI, 1.20–2.13; *P* < .01) in the HADS-depression scale, 2.17 points (95% CI, 1.81–2.53; *P* < .01) in the PROMIS-10 mental health domain and 1.31 points (95% CI, 0.96–1.66; *P* < .01) in the PROMIS-10 physical health domain (Figure 2). Detailed measures of PROMs assessed for each cohort are presented in Table 4.

**Table 4:** Main PROMs in the historic and HARMONICS-HVH cohorts after propensity score matching.

**PROMs at 90 days**	**Historic cohort (*n* = 882)**	**HARMONICS-HVH (*n* = 882)**	**Mean difference (95% CI)**	***P*-value**
**Quality of life (0–100) (median, IQR)**	70.00 (50.00, 80.00)	70.00 (50.00, 82.00)	−2.4274 (−4.4810, −0.3738)	.0205
**HADS anxiety (median, IQR)**	6.00 (3.00, 9.00)	4.00 (2.00, 7.00)	1.2225 (0.8100,1.6350)	<.0001
**HADS depression (median, IQR)**	5.00 (1.00, 9.00)	3.00 (1.00, 7.00)	1.6386 (1.1742, 2.1030)	<.0001
**PROMIS-10-M (median, IQR)**	11.00 (8.00, 13.00)	13.00 (11.00, 15.00)	−2.1925 (−2.5517, −1.8334)	<.0001
**PROMIS-10-PHY (median, IQR)**	13.00 (11.00, 16.00)	15.00 (12.00, 17.00)	−1.2080 (−1.5558, −0.8602)	<.0001

Abbreviations: HADS=Hospital Anxiety and Depression Scale; HVH=Hospital Universitari Vall d’Hebron; PROMIS-10-M=Patient Reported Outcomes Measurement Information System-mental health domain (10-item scale); PROMIS-10-PHY=Patient Reported Outcomes Measurement Information System-physical health domain (10-item scale); PROMs=patient-reported outcomes.

## Discussion

This manuscript describes the design, implementation and principal outcomes of the HARMONICS plan, a multicentre initiative aimed at harmonising workflows, enhancing communication and delivering value-based healthcare to stroke survivors after admission to 6 CSCs in Spain and Portugal. Standardised workflows were co-developed across centres, and the plan was progressively implemented during 2022–2023, concluding recruitment in November 2024. A total of 4209 acute stroke patients were enrolled, representing 84% of those meeting inclusion criteria.

The primary objective was to implement the HARMONICS plan across participating centres while achieving predefined feasibility thresholds. Only 36% of stroke patients admitted to the 6 CSCs during the recruitment period were eligible for the study. HARMONICS was designed with the aim of being as inclusive as possible to increase its impact on post-stroke care. However, the study was conducted in CSCs that receive a high proportion of transferred patients from non-directly covered areas, and the CSCs do not provide the follow-up for them, constituting one of the main limitations for recruitment. Multiple clinical studies and trials were conducted in parallel with HARMONICS in the different CSC, also impacting recruitment. However, the main limitation falls in the expected inability for the follow-up. CSCs receive patients with higher stroke severity candidates to reperfusion treatment evaluations and may have higher rates of mRS of 5 at discharge than non-CSCs, being these patients directly excluded from the study. In addition, patients with an mRS 3–4 in our healthcare system are frequently transferred to nursing homes with expected prolonged stays. These patients cannot perform HARMONICS follow-up by themselves, and families in this situation are reluctant to participate in a follow-up study if they are not being the direct caregivers of the patients. Even with these exclusions, there was still a high rate of loss to follow-up, and those initially using the app changed to telephone-based follow-up in almost 20% of cases. Patient-reported outcomes collection was poor: at 90 days, 53% of patients completed PROMs, decreasing to 34.2% at 1 year, similar to retention rates in historical controls, underscoring persistent challenges in long-term data capture. Therefore, even though the feasibility indicators were largely met, the success of the implementation can only be considered partial.

Patient satisfaction was high at 90 days (median score 10), with 72% willing to recommend the programme and 84% retained. Nevertheless, nearly half of participants were lost to follow-up by 1 year, predominantly those with milder strokes using the app independently. This suggests lower perceived disease severity and the need for enhanced engagement strategies.^29^ During hospitalisation, CMs introduced the plan, highlighting the direct communication channel after discharge. Only 5% declined participation. Initial engagement was high—80% of patients used chat consultations—and educational materials were widely accessed, likely improving health literacy and self-management.

However, sustaining engagement proved challenging. Forty percent of patients preferred phone follow-up from the outset; among the 60% choosing the app, half relied on a caregiver for installation. At 90 days, 20% had switched to phone contact, indicating usability issues or preference for direct communication. The rate of PROMs collection at 90 days, however, was similar between app or phone users. The median age was 73 years, and most caregivers were similarly aged spouses, reflecting persistent barriers to technology adoption among older adults. Although smartphone usage is on the rise, barriers to technology adoption remain prevalent among the elderly population, which suggests that only younger patients with milder strokes may currently embrace an app-based follow-up approach. Nevertheless, it is anticipated that this landscape will evolve over the next few years.^30^

Post-stroke cognitive deficits may also limit engagement. Stroke survivors may experience cognitive deficits that go unrecognised immediately after the event but become apparent in subsequent weeks when they return home.^31^ Aphasia was not considered an exclusion criterion in our study but given the low median NIHSS and mRS of our series, few patients with disabling aphasia were recruited in the study and its impact on the engagement difficulties is probably low. Nevertheless, these factors, coupled with the previously noted high rates of loss to follow-up, present significant challenges in ensuring that PROMs are consistently collected and that long-term follow-up is achieved. Most educational materials were delivered within the first month, when the risk of recurrence is high, but potentially contributing to declining use of the app over time. Patient-reported outcomes collection, requiring lengthy surveys, may have been perceived as burdensome. Alerts generated from pathological scores prompted CM interventions, but many patients—even with mild strokes—reported persistently poor PROMs, suggesting unresolved health needs.^13^ Our study reflects a reality that has been detected with the implementation of value-based healthcare approaches: the self-perception of health status and quality of life after stroke are poor, even in patients with favourable mRS. The mRS may be insufficient to capture the complexity of the different spheres of health that matter to patients, and the systematic collection of PROMs in chronic diseases is key to improving their care. However, we detected a lower-than-previously reported rate of post-stroke anxiety and depression in our series, which may be related to specific characteristics of our population, or the tool used for their measurement.

Pre-implementation workflow assessment using lean methodology identified communication gaps between patients and healthcare professionals, and among professionals from different providers. Social and functional assessments were inconsistently performed, and post-discharge coordination often depended on patients or relatives. Addressing these challenges, including the lack of workflow coordination, necessitates a multidisciplinary and multicentre intervention that encompasses all healthcare institutions partaking in the patient’s stroke care pathway. In HARMONICS, the CM role was introduced to address these gaps, coordinating care and acting as a liaison via app or phone. However, interventions may not have fully addressed patient needs, contributing to early attrition.

Implementation varied across centres, influenced by recruitment duration and competing studies. The centre with highest penetration had prior experience with value-based programmes. Adopting value-based healthcare entails cultural and procedural change, which may slow uptake and face resistance.^32^

For historical comparisons, only patients from the experienced centre with available pre-HARMONICS outcomes (including PROMs) were analysed. No differences in mortality were observed, likely reflecting the relatively young age and mild stroke severity of the recruited patients. Propensity score matching on key prognostic variables showed improvements in PROMs and 3-month functional status after HARMONICS implementation, while recurrence rates remained unchanged. This pattern suggests that the programme primarily enhanced continuity of care and early detection of post-stroke complications through closer follow-up, rather than directly influencing vascular risk factor control or long-term treatment adherence. The historical cohort (2019–2021) preceded the programme’s launch in February 2022, with no other major changes in post-hospital stroke care, supporting the hypothesis that improved communication and education contributed to the observed benefits. Nevertheless, unmeasured factors cannot be excluded.

Study limitations include the pilot, non-randomised design and use of historical controls from a single centre. In addition, the feasibility thresholds evaluating the main outcome of the study were arbitrarily defined. Although patient profiles were comparable before and during implementation, and propensity adjustment was applied, generalisability is limited. The cohort was largely composed of young patients with mild strokes, with severe strokes, language barriers and transfers excluded, meaning our results may not be generalisable to the elderly and severe stroke population. In addition, almost 50% of patients were recruited in 1 single centre with experience on value-based healthcare programmes, what can again affect the generalisability of our results. Initial follow-up modality influenced engagement; whether exclusive app use would have improved outcomes remains unknown. Addressing the digital divide is essential for equitable participation in digital health, yet remains challenging, particularly for older or cognitively impaired patients.^33^

Finally, substantial loss to follow-up may have biased our results, as non-responders could differ systematically from responders. This may partly explain the relatively high 1-year recurrence and mortality rates observed in our quite young cohort with mild stroke severity; deaths and recurrences are reliably captured in our electronic medical records, which may result in an overrepresentation of these adverse outcomes. Collecting PROMs and ensuring sustained follow-up for stroke patients remains a formidable challenge, one that we attempted to mitigate through the HARMONICS plan, albeit with only partial success.

In conclusion, HARMONICS, as a value-based healthcare model, places patients at the centre of care, promoting education, engagement and self-responsibility. Systematic collection of CROMs and PROMs across centres fosters a collaborative framework for benchmarking and quality improvement. Lean methodology revealed deficiencies in post-hospital workflows, and HARMONICS introduced partial solutions. Implementation across 6 CSCs in 2 countries demonstrated feasibility, with high rates of satisfaction reported by patients. Greater integration with primary care and enhanced inter-provider communication may further increase benefits. A stepped-wedge cluster randomised trial should be considered to rigorously evaluate the model’s effectiveness in improving post-stroke outcomes.

## Supplementary Material

aakag016_SUPPLEMENTAL_MATERIAL_HARMONICS_V2
