# Supplementary material for: HARMONICS: feasibility of a holistic value-based care hybrid programme that maximises clinical outcomes after stroke
Source: Eur Stroke J. 2026 Mar 17;11(3):aakag016. doi: 10.1093/esj/aakag016 (PMC12994693; doi:10.1093/esj/aakag016)
Supplement: aakag016_SUPPLEMENTAL_MATERIAL_HARMONICS_V2 [file aakag016_supplemental_material_harmonics_v2.pdf]

SUPPLEMENTAL MATERIAL HARMONICS

Supplemental Table S1: CONSORT Checklist

CONSORT 2010 checklist of information to include when reporting a pilot or feasibility trial*

Section/Topic	Item No	Checklist item	Reported on page No
**Title and abstract**			
	1a	Identification as a pilot or feasibility randomised trial in the title	1 (non-randomized)
	1b	Structured summary of pilot trial design, methods, results, and conclusions (for specific guidance see CONSORT abstract extension for pilot trials)	3
**Introduction**			
Background and objectives	2a	Scientific background and explanation of rationale for future definitive trial, and reasons for randomised pilot trial	6, 34
	2b	Specific objectives or research questions for pilot trial	9-10
**Methods**			
Trial design	3a	Description of pilot trial design (such as parallel, factorial) including allocation ratio	7-8 (non-randomized)
	3b	Important changes to methods after pilot trial commencement (such as eligibility criteria), with reasons	N.A
Participants	4a	Eligibility criteria for participants	8-9
	4b	Settings and locations where the data were collected	7, Figure 1
	4c	How participants were identified and consented	8
Interventions	5	The interventions for each group with sufficient details to allow replication, including how and when they were actually administered	14-16 (non-randomized)
Outcomes	6a	Completely defined prespecified assessments or measurements to address each pilot trial objective specified in 2b, including how and when they were assessed	9
	6b	Any changes to pilot trial assessments or measurements after the pilot trial commenced, with reasons	N.A
	6c	If applicable, prespecified criteria used to judge whether, or how, to proceed with future definitive trial	N.A
Sample size	7a	Rationale for numbers in the pilot trial	12-13
	7b	When applicable, explanation of any interim analyses and stopping guidelines	N.A.
Randomisation:			
Sequence	8a	Method used to generate the random allocation sequence	N.A

generation	8b	Type of randomisation(s); details of any restriction (such as blocking and block size)	N.A
Allocation concealment mechanism	9	Mechanism used to implement the random allocation sequence (such as sequentially numbered containers), describing any steps taken to conceal the sequence until interventions were assigned	N.A
Implementation	10	Who generated the random allocation sequence, who enrolled participants, and who assigned participants to interventions	N.A
Blinding	11a	If done, who was blinded after assignment to interventions (for example, participants, care providers, those assessing outcomes) and how	N.A
	11b	If relevant, description of the similarity of interventions	N.A
Statistical methods	12	Methods used to address each pilot trial objective whether qualitative or quantitative	12-13
**Results**			
Participant flow (a diagram is strongly recommended)	13a	For each group, the numbers of participants who were approached and/or assessed for eligibility, randomly assigned, received intended treatment, and were assessed for each objective	16-17, Fig. 1
	13b	For each group, losses and exclusions after randomisation, together with reasons	Fig 1
Recruitment	14a	Dates defining the periods of recruitment and follow-up	16
	14b	Why the pilot trial ended or was stopped	16
Baseline data	15	A table showing baseline demographic and clinical characteristics for each group	Table 2
Numbers analysed	16	For each objective, number of participants (denominator) included in each analysis. If relevant, these numbers should be by randomised group	18, 27<br>Table 2 and 3
Outcomes and estimation	17	For each objective, results including expressions of uncertainty (such as 95% confidence interval) for any estimates. If relevant, these results should be by randomised group	N.A
Ancillary analyses	18	Results of any other analyses performed that could be used to inform the future definitive trial	27-28, Table 4
Harms	19	All important harms or unintended effects in each group (for specific guidance see CONSORT for harms)	N.A
	19a	If relevant, other important unintended consequences	N.A
**Discussion**			
Limitations	20	Pilot trial limitations, addressing sources of potential bias and remaining uncertainty about feasibility	29-30, 33
Generalisability	21	Generalisability (applicability) of pilot trial methods and findings to future definitive trial and other studies	33
Interpretation	22	Interpretation consistent with pilot trial objectives and findings, balancing potential benefits and harms, and considering other relevant evidence	29-34
	22a	Implications for progression from pilot to future definitive trial, including any proposed amendments	34
**Other information**			
Registration	23	Registration number for pilot trial and name of trial registry	N.A.
Protocol	24	Where the pilot trial protocol can be accessed, if available	N.A
Funding	25	Sources of funding and other support (such as supply of drugs), role of funders	7, 34

	26	Ethical approval or approval by research review committee, confirmed with reference number	7

Citation: Eldridge SM, Chan CL, Campbell MJ, Bond CM, Hopewell S, Thabane L, et al. CONSORT 2010 statement: extension to randomised pilot and feasibility trials. BMJ. 2016;355. This is an Open Access article distributed in accordance with the terms of the Creative Commons Attribution (CC BY 3.0) license (http://creativecommons.org/licenses/by/3.0/), which permits others to distribute, remix, adapt and build upon this work, for commercial use, provided the original work is properly cited.

*We strongly recommend reading this statement in conjunction with the CONSORT 2010, extension to randomised pilot and feasibility trials, Explanation and Elaboration for important clarifications on all the items. If relevant, we also recommend reading CONSORT extensions for cluster randomised trials, non-inferiority and equivalence trials, non-pharmacological treatments, herbal interventions, and pragmatic trials. Additional extensions are forthcoming: for those and for up-to-date references relevant to this checklist, see www.consort-statement.org.

Supplemental Table S2: STROBE checklist

Section	Item	Description	Notes	Pag.
**Introduction**	2	Explain the scientific background and rationale for the investigation being reported		5-6
	3	State specific objectives, including any prespecified hypotheses		9-10
**Methods**	4	Present key elements of study design early in the paper	Study Design	7-8
	5	Describe the setting, locations, and relevant dates, including periods of recruitment, exposure, follow-up, and data collection	Setting	7,16
	6	(a) Describe eligibility, selection methods, and follow-up methods depending on study type (cohort, case-control, cross-sectional)	Participants	8-9
		(b) Describe matching criteria and numbers for matched studies		13
	7	Clearly define all outcomes, exposures, predictors, potential confounders, and effect modifiers; give diagnostic criteria if applicable	Variables	9-10
	8	For each variable, give data sources and assessment methods; describe comparability if multiple groups exist	Data Sources/Measurement	10
	9	Describe any efforts to address potential sources of bias	Bias	11
	10	Explain how the study size was determined	Study Size	12-13,N.A
	11	Explain handling of quantitative variables; justify groupings	Quantitative Variables	9, Table 1
	12	(a) Describe statistical methods including confounder control	Statistical Methods	12-13
		(b) Describe subgroup and interaction methods		N.A
		(c) Explain missing data handling		13
		(d) Cohort: address loss to follow-up; Case-control: matching; Cross-sectional: sampling strategy		12-13
		(e) Describe any sensitivity analyses		N.A
**Results**	13	(a) Report participant flow through study stages	Participants	16, Fig. 1

Section	Item	Description	Notes	Pag.
		(b) Give reasons for non-participation		8-9, Fig.1
		(c) Consider using a flow diagram		Fig. 1
	14	(a) Describe participant characteristics and confounders	Descriptive Data	18, 21, Table 2
		(b) Indicate missing data per variable		Table 2
		(c) Cohort: summarise follow-up time		23-24
	15	Cohort: outcome events over time; Case-control: exposure category data; Cross-sectional: outcome data	Outcome Data	24-26, Table 3
	16	Report main results including adjusted estimates and precision	Main Results	24-26
	17	Report other analyses (e.g., subgroups, interactions, sensitivity)	Other Analyses	26-28, Table 4
**Discussion**	18	Summarise key results in relation to objectives	Key Results	29-32
	19	Discuss limitations, including direction and magnitude of bias	Limitations	33
	20	Interpret results cautiously, considering all aspects	Interpretation	29-34
	21	Discuss generalisability of the findings	Generalizability	33
**Other Information**	22	Report funding sources and roles of funders	Funding	7, 34

Supplemental Table S3: Dimensions measured through patient- and clinician reported outcomes, pre-defined key performance indicators of the HARMONICS plan and patients reported experience measurement**.** C: clinician-reported outcomes. P: patients reported outcomes

Dimensions	Outcome description	Outcome measurement	Timing of collection
Survival	Survival	C: Rate of modified Rankin Scale (mRS) <6	3 months, 1 year
Recurrence	New stroke occurrence	C: rate of recurrence	1 year
Functional recovery	Functional status	C: mRS	3 months, 1 year
Quality of health	Self-perceived quality of health	P: EQ5-Visual 0-100 scale of quality of health	3 months, 1 year
Physical health self-perception	Self-perceived physical status	P: PROMIS 10 physical dimension	3 months, 1 year
Mental health self-perception	Self-perceived mental status	P: PROMIS 10 mental dimension<br>P: HAD anxiety and depression	3 months, 1 year<br>3 months, 1 year
Treatment adherence	Adherence to pharmacological treatment	P: Morisky Green scale	3 months, 1 year
**KPI**	**KPI description**	**KPI measurement**	**Favorable KPI**

Stroke inclusion rate	Patients included in HARMONICS plan	Rate of hospitalized stroke patients fulfilling criteria included in HARMONICS	>60%
Retention within the HARMONICS plan	Patients showing interaction with the case managers through the NORA app	Retention within the HARMONICS plan, measured as the proportion of patients contacting the CM (≥1 chats) and/or answering the surveys for PROMS collection in patients followed by the NORA app and/or maintaining follow-up by NORA after 3 months from acute stroke	>75%
PROMs recollection rate	HARMONICS patients answering PROMs surveys	Rate of surveys fulfilment	>60%
**PREM**	**PREM description**	**PREM measurement**	**Favorable PREM**
Satisfaction with HARMONICS plan	Patient perception of the usability of the technological tool and the holistic follow-up	Picker-type scale of recommendation of the HARMONICS plan to a family or friend suffering a stroke	9-10 score in >70% of patients

Supplemental Table S4: Baseline covariates before and after propensity score matching of the historical cohort and the HARMONICS cohort with available CROMs (mRs) at 90 days.

Variable	Pre-HARMONICS (N=2160)	HARMONICS (N=1475)	SD	Pre-HARMONICS (N=1433)	HARMONICS(N=1433)	SD
**Age**	73.00 (62.00, 82.00)	74.00 (62.00, 82.00)	0.07	74.00 (63.00, 83.00)	74.00 (62.00, 82.00)	<0.01
**Sex (Female)**	954 (44.2%)	640 (43.4%)	0.01	623 (43.5%)	621 (43.3%)	0.02
**NIHSS**	3.00 (1.00, 7.00)	3.00 (1.00, 7.00)	0.03	3.00 (1.00, 7.00)	3.00 (1.00, 7.00)	0.01
**Wake-up Stroke or Uncertain Onset**	624 (28.9%)	443 (30.0%)	0.02	422 (29.4%)	425 (29.7%)	0.02
**Baseline mRS**						
0	746 (34.5%)	496 (33.6%)	0.02	487 (34.0%)	489 (34.1%)	0.03
1	813 (37.6%)	498 (33.8%)	0.08	471 (32.9%)	488 (34.1%)	0.04
2	332 (15.4%)	274 (18.6%)	0.09	268 (18.7%)	257 (17.9%)	0.01
3	223 (10.3%)	170 (11.5%)	0.04	170 (11.9%)	166 (11.6%)	0.01
4	46 (2.1%)	37 (2.5%)	0.03	37 (2.6%)	33 (2.3%)	0.02
**Stroke Diagnosis**						

TIA	247 (11.4%)	190 (12.9%)	0.04	196 (13.7%)	189 (13.2%)	0.03
ICH	168 (7.8%)	112 (7.6%)	<0.01	106 (7.4%)	107 (7.5%)	<0.01
Ischemic Stroke	1714 (79.4%)	1134 (76.9%)	0.06	1110 (77.5%)	1115 (77.8%)	0.03
Other	31 (1.4%)	39 (2.6%)	0.11	21 (1.5%)	22 (1.6%)	0.02
**Hypertension**	781 (36.2%)	517 (35.1%)	0.02	502 (35.0%)	497 (34.7%)	<0.01
**Dyslipidemia**	927 (42.9%)	616 (41.8%)	0.02	588 (41.0%)	592 (41.3%)	0.03
**Diabetes Mellitus Type 2**	347 (16.1%)	214 (14.5%)	0.04	203 (14.2%)	203 (14.2%)	0.01
**Heart Failure**	92 (4.3%)	124 (8.4%)	0.2	89 (6.2%)	102 (7.1%)	0.07
**Smoking**	649 (30.0%)	456 (30.9%)	0.02	431 (30.1%)	437 (30.5%)	0.02
**Atrial fibrillation**	293 (13.6%)	198 (13.4%	<0.01	188 (13.1%)	187 (13.0%)	0.02
**Ischemic cardiopathy**	236 (10.9%)	165 (11.2%	<0.01	162 (11.3%)	156 (10.9%)	<0.01
**EVT**	410 (19.0%)	296 (20.1%)	0.03	284 (19.8%)	289 (20.2%)	0.02
**IVT**	313 (14.5%)	237 (16.1%)	0.04	228 (15.9%)	227 (15.8%)	0.01
**Discharge destination home**	1819 (84.2%)	1257 (85.2%)	0.03	1215 (84.8%)	1224 (85.4%)	0.01
**NIHSS at discharge**	1.00 (0.00, 3.00)	1.00 (0.00, 2.00)	0.09	1.00 (0.00, 2.00)	1.00 (0.00, 2.00)	0.02
**Modified Rankin Scale at Discharge**						
0	243 (11.2%)	233 (15.8%)	0.14	217 (15.1%)	228 (15.9%)	0.04
1	627 (29.0%)	435 (29.5%)	0.01	429 (29.9%)	426 (29.7%)	<0.01

2	513 (23.8%)	357 (24.2%)	0.01	347 (24.2%)	341 (23.8%)	<0.01
3	494 (22.9%)	288 (19.5%)	0.08	272 (19.0%)	283 (19.7%)	0.03
4	283 (13.1%)	162 (11.0%)	0.06	168 (11.7%)	155 (10.8%)	<0.01

Supplemental Table S5: Baseline covariates before and after propensity score matching of the historical cohort and the HARMONICS cohort with available PROMs at 90 days

Variable	Pre-HARMONICS (N=1076)	HARMONICS (N=963)	SD	Pre-HARMONICS (N=886)	HARMONICS (N=886)	SD
**Age**	74.00 (61.00, 82.00)	73.00 (62.00, 82.00)	0.01	74.00 (61.00, 82.00)	73.00 (62.00, 81.75)	0.01
**Sex (Female)**	479 (44.5%)	408 (42.4%)	0.04	389 (43.9%)	379 (42.8%)	0.02
**NIHSS**	3.00 (1.00, 7.00)	3.00 (1.00, 6.00)	0.06	3.00 (1.00, 7.00)	3.00 (1.00, 6.00)	0.03
**Wake-up Stroke or Uncertain Onset**	300 (27.9%)	289 (30.0%)	0.05	247 (27.9%)	257 (29.0%)	0.02
**Baseline mRS**						
0	406 (37.7%)	346 (35.9%)	0.04	330 (37.2%)	326 (36.8%)	<0.01
1	383 (35.6%)	325 (33.7%)	0.04	306 (34.5%)	304 (34.3%)	<0.01
2	156 (14.5%)	184 (19.1%)	0.13	144 (16.3%)	159 (17.9%)	0.05
3	115 (10.7%)	88 (9.1%)	0.05	90 (10.2%)	81 (9.1%)	0.03
4	16 (1.5%)	20 (2.1%)	0.05	16 (1.8%)	16 (1.8%)	<0.01

**Stroke Diagnosis**						
TIA	136 (12.6%)	140 (14.5%)	0.06	126 (14.2%)	129 (14.6%)	0.01
ICH	93 (8.6%)	62 (6.4%)	0.08	57 (6.4%)	61 (6.9%)	0.02
Ischemic Stroke	825 (76.7%)	734 (76.2%)	0.01	685 (77.3%)	678 (76.5%)	0.02
Other	22 (2.1%)	27 (2.9%)	0.05	18 (2.1%)	18 (2.1%)	<0.01
**Hypertension**	380 (35.3%)	320 (33.2%)	0.04	291 (32.8%)	297 (33.5%)	0.01
**Dyslipidemia**	462 (42.9%)	403 (41.8%)	0.02	373 (42.1%)	367 (41.4%)	0.01
**Diabetes Mellitus Type 2**	462 (42.9%)	133 (13.8%)	0.02	111 (12.5%)	123 (13.9%)	0.04
**Heart Failure**	40 (3.7%)	76 (7.9%)	0.22	39 (4.4%)	39 (4.4%)	<0.01
**Smoking**	317 (29.5%)	302 (31.4%)	0.04	275 (31.0%)	277 (31.3%)	<0.01
**Atrial fibrillation**	136 (12.6%)	122 (12.7%)	<0.01	109 (12.3%)	104 (11.7%)	0.02
**Ischemic cardiopathy**	110 (10.2%)	104 (10.8%)	0.02	83 (9.4%)	89 (10.0%)	0.02
**Antiplatelet therapy**	196 (18.2%)	165 (17.1%)	0.03	159 (17.9%)	156 (17.6%)	<0.01
**Anticoagulation therapy**	55 (5.1%)	60 (6.2%)	0.05	49 (5.5%)	49 (5.5%)	<0.01
**EVT**	209 (19.4%)	186 (19.3%)	<0.01	169 (19.1%)	167 (18.8%)	<0.01
**IVT**	175 (16.3%)	173 (18.0%)	0.04	159 (17.9%)	153 (17.3%)	0.02
**Discharge destination home**	935 (86.9%)	840 (87.2%)	<0.01	781 (88.1%)	778 (87.8%)	<0.01

**NIHSS at discharge**	1.00 (0.00, 2.00)	0.00 (0.00, 2.00)	0.13	1.00 (0.00, 2.00)	1.00 (0.00, 2.00)	0.03
**Modified Rankin Scale at Discharge**						
0	133 (12.4%)	168 (17.4%)	0.15	133 (15.0%)	147 (16.6%)	0.04
1	322 (29.9%)	303 (31.5%)	0.03	292 (33.0%)	286 (32.3%)	0.01
2	266 (24.7%)	235 (24.4%)	<0.01	210 (23.7%)	212 (23.9%)	<0.01
3	249 (23.1%)	162 (16.8%)	0.15	166 (18.7%)	155 (17.5%)	0.03
4	106 (9.9%)	95 (9.9%)	<0.01	85 (9.6%)	86 (9.7%)	<0.01

Supplemental figure S1: Technological solution of the HARMONICS program, NORA. a) mobile application for patients/caregivers, b) web-platform for case managers, c) dashboard for study monitoring and benchmarking.

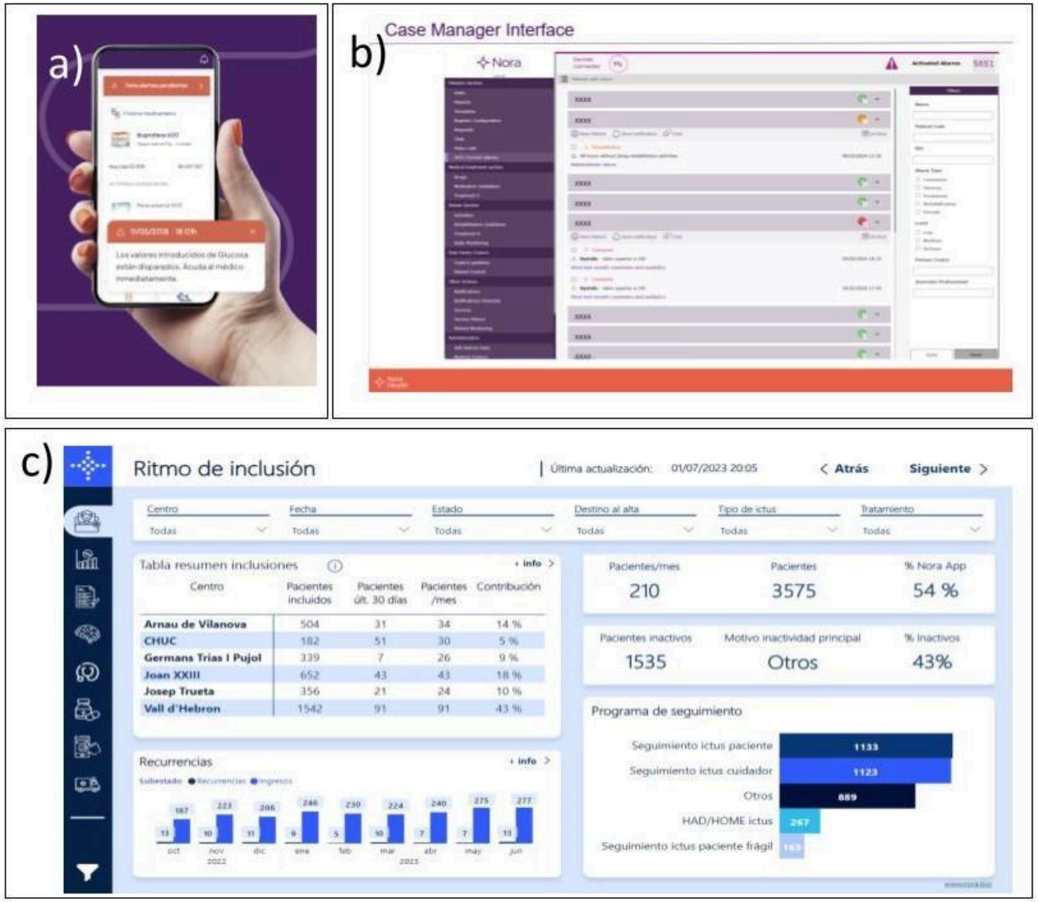

HARMONICS INVESTIGATORS

Alsina, M. Dolors, Gerencia Atenció Primaria, Girona

Alba, Cristina, Gerencia Atenció Primaria Barcelona Nord

Avelli, Judith, Gerencia Atenció Primaria, Girona

Avivar, Ylènia, Hospital Universitari Joan XXIII, Tarragona

Ayesta, Mercedes, NORA Health, Barcelona

Baladas, Maria, Hospital Universitari Vall d'Hebron, Barcelona

Barta, Laura, Hospital Universitari Arnau de Vilanova, Lleida

Bernardo-Castro, Sara, Unidade Local de Saúde de Coimbra, Portugal

Bustamante, Alejandro, Hospital Universitari Germans Trias i Pujol, Badalona

Canela, Noelia, Hospital Universitari Joan XXIII, Tarragona

Cano, David, NORA Health, Barcelona

Colangelo, Giorgio, Hospital Universitari Vall d'Hebron, Barcelona

Dolz, Elia, Hospital Universitari Germans Trias i Pujol, Badalona

Elizondo, Olman A., Agencia Qualitat i Evaluació Sanitaria (AQUAS), Barcelona

Fernandes, Catarina, Unidade Local de Saúde de Coimbra, Portugal

Ferreira, Ana Margarida, Unidade Local de Saúde de Coimbra, Portugal

Flores, Alan, Hospital Universitari Joan XXIII, Tarragona

Franco, María, Hospital Universitari Vall d'Hebron, Barcelon

Garcia-Tornel, Alvaro, Hospital Universitari Vall d'Hebron, Barcelona

Guirao, Cristina, Hospital Universitari Vall d'Hebron, Barcelona

Mauri, Gerard, Hospital Universitari Arnau de Vilanova, Lleida

Millán, Mònica, Hospital Universitari Germans Trias i Pujol, Badalona

Molleda, Mercè, Hospital Universitari Germans Trias i Pujol, Badalona

Molina, Carlos A., Hospital Universitari Vall d'Hebron, Barcelona

Moran, Estefanía, Roche España, Barcelona

Muchada, Marian, Hospital Universitari Vall d'Hebron, Barcelona

Olivé, Marta, Hospital Universitari Vall d'Hebron, Barcelona

Ortiz, Elisabeth, Hospital Universitari Doctor Josep Trueta, Girona

Pagola, Jorge, Hospital Universitari Vall d'Hebron, Barcelona

Paredes, Carolina, Hospital Universitari Vall d'Hebron, Barcelona

Pérez de la Ossa, Natàlia, Hospital Universitari Sant Pau

Purroy, Francisco, Hospital Universitari Arnau de Vilanova, Lleida

Ribo, Marc, Hospital Universitari Vall d'Hebron, Barcelona

Rizzo, Federica, Hospital Universitari Vall d'Hebron, Barcelona

Rodriguez-Luna, David, Hospital Universitari Vall d'Hebron, Barcelona

Rodríguez-Villatoro, Noelia, Hospital Universitari Vall d'Hebron, Barcelona

Rubiera, Marta, Hospital Universitari Vall d'Hebron, Barcelona

Sanchez-Gabilan, Esther, Hospital Universitari Vall d'Hebron, Barcelona

Sargento-Freitas, João, Unidade Local de Saúde de Coimbra, Portugal

Seró, Laia, Hospital Universitari Joan XXIII, Tarragona

Silva, Fernando, Unidade Local de Saúde de Coimbra, Portugal

Silva, Yolanda, Hospital Universitari Doctor Josep Trueta, Girona

Simonetti, Renato, Hospital Universitari Vall d'Hebron, Barcelona

Soria, Sònia, Hospital Universitari Germans Trias i Pujol, Badalona

Ustrell, Xavier, Hospital Universitari Joan XXIII, Tarragona

Valls, Victoria, Agencia Qualitat i Evaluació Sanitaria (AQUAS), Barcelona

Vioquez, María, Gerencia Atenció Primaria Barcelona Nord
